# Indiana in Want of a Medical Journal. (?)

**Published:** 1868-11

**Authors:** 


### Indiana in Want of a Medical Journal. (?)

Dr. J. S. Botts, of Indianapolis, President of Indiana State Medical Society, quotes in his address, the report of a committee, complaining of the want of a proper medium of communication for members of the medical profession. "If our medical brethren, throughout the length and breadth of the State, will consent to communicate the result of their observations, freely, we feel assured that a Medical Journal, of a very high order of usefulness, can be sustained, which will prove not only a medium of intelligence, but also a bond of union amongst medical practitioners. Impressed by the belief that such will be the results, we would respectfully recommend the establishment of a journal immediately." This looks as though Dr. Botts and the Committee must "live in France and never hear of Napoleon." In the very city where Dr. Botts lives, is published one of the very best conducted, and most valuable medical journals in the country—the *Western Journal of Medicine*, edited by one of the most, if not *the* most, intelligent and worthy physician of the State. As now conducted, it is well worthy the support of the profession. That we could say as much of it, if contributed to by Dr. Botts and the Committee, is a matter of great doubt; judging from the spirit of the address and report, we could not. We have never seen any other of Dr. Bott's contributions to medical literature, or anything from any member of the Committee; if any have been published, we should be glad to receive a copy. It must have been through some very choice "medium." When will members of the profession learn to control their prejudices, and not exhibit their worst qualities upon the most inappropriate occasions?

